# Disulfiram Protects Against Radiation-Induced Intestinal Injury in Mice

**DOI:** 10.3389/fphar.2022.852669

**Published:** 2022-04-19

**Authors:** Qingwen Yuan, Renjun Peng, Huijie Yu, Sinian Wang, Zhongmin Chen, Suhe Dong, Wei Li, Bo Cheng, Qisheng Jiang, Yuwen Cong, Fengsheng Li, Changzheng Li

**Affiliations:** ^1^ The Postgraduate Training Base of Jinzhou Medical University, The PLA Rocket Force Characteristic Medical Center, Beijing, China; ^2^ Department of Nuclear Radiation Injury and Monitoring, The PLA Rocket Force Characteristic Medical Center, Beijing, China; ^3^ Department of Pathology, The PLA Rocket Force Characteristic Medical Center, Beijing, China; ^4^ Department of Pathophysiology, Beijing Institute of Radiation Medicine, Beijing Key Laboratory for Radiobiology (BKLRB), Beijing, China; ^5^ Department of Gastroenterology, The PLA Rocket Force Characteristic Medical Center, Beijing, China

**Keywords:** disulfiram, radioprotector, radiation-induced intestinal injury, Lgr5+ stem cell, DNA damage

## Abstract

Radiation-induced intestinal injury (RIII) occurs after high doses of radiation exposure. RIII restricts the therapeutic efficacy of radiotherapy in cancer and increases morbidity and mortality in nuclear disasters. Currently, there is no approved agent for the prevention or treatment of RIII. Here, we reported that the disulfiram, an FDA-approved alcohol deterrent, prolonged the survival in mice after lethal irradiation. Pretreatment with disulfiram inhibited proliferation within 24 h after irradiation, but improved crypt regeneration at 3.5 days post-irradiation. Mechanistically, disulfiram promoted Lgr5^+^ intestinal stem cells (ISCs) survival and maintained their ability to regenerate intestinal epithelium after radiation. Moreover, disulfiram suppresses DNA damage accumulation, thus inhibits aberrant mitosis after radiation. Unexpectedly, disulfiram treatment did not inhibit crypt cell apoptosis 4 h after radiation and the regeneration of crypts from PUMA-deficient mice after irradiation was also promoted by disulfiram. In conclusion, our findings demonstrate that disulfiram regulates the DNA damage response and survival of ISCs through affecting the cell cycle. Given its radioprotective efficacy and decades of application in humans, disulfiram is a promising candidate to prevent RIII in cancer therapy and nuclear accident.

## Introduction

The bone marrow and the small intestine are radiosensitive tissues, and a high dose of radiation results in hematopoietic and intestinal injury (McBride and Schaue, 2020). Radiation-induced intestinal injury (RIII) manifests following whole-body or abdominal irradiation at a dose of more than 6 Gy in humans. At these doses of radiation, massive intestinal stem cells (ISCs) are depleted, which compromises epithelial integrity and impairs epithelial renewal. The breakdown of the mucosal barrier further leads to fluid loss, electrolyte imbalance, sepsis and even death (Macià I Garau et al., 2011). In contrast to hematopoietic failure that can be rescued by bone marrow transplantation and supportive care, there are no approved agents to prevent or mitigate RIII (Singh and Seed, 2017). Importantly, despite advances in treatment delivery techniques, abdominal and pelvic radiotherapy inevitably evoke intestinal toxicity that restricts maximum doses of irradiation, which prevents patients from competing treatment and causes acute and chronic gastrointestinal tract complications, thus limiting the efficiency of therapy and reducing the patient’s quality of life (Harb et al., 2014). Therefore, highly effective radioprotectors with fewer side effects are urgently needed for RIII.

ISCs reside at the bottom of the crypts and differentiate into highly proliferative transit amplifying cells that give rise to all differentiated cell types, including enterocytes, goblet cells, tuft cells, enteroendocrine cells and Paneth cells. Thus, ISCs are fundamental to the maintenance of intestinal homeostasis and epithelia regeneration following radiation exposure (Barker et al., 2012). Ionizing radiation (IR) induces DNA damage, and DNA double-strand break (DSB) is the most serious type of DNA damage. DSBs in ISCs and intestinal progenitors initiate a coordinated signaling network that recognizes exposed DNA lesions, induces cell cycle arrest, activates DSB repair or signals cell death (Lund, 2012). Various modes of radiation-induced intestinal cell death, including apoptosis, mitotic catastrophe and pyroptosis have been implicated (Kirsch et al., 2010; Leibowitz et al., 2011; Hu et al., 2016). Accumulating evidence shows that IR-induced gastrointestinal epithelium apoptosis occurs in two distinct phases. Radiation exposure rapidly activates p53 in crypt cells, which initiates p53-upregulated modulator of apoptosis-dependent (PUMA-dependent) apoptosis *via* the intrinsic pathway within 3–6 h following radiation. A delayed wave of p53-independent apoptosis occurs at 24 h after irradiation when ISCs and crypt progenitors are released from G2 arrest (Kirsch et al., 2010). Unsolved DSBs in proliferative crypt cells render aberrant mitosis or mitotic catastrophe that compromises epithelium regeneration. Researches indicate that the delayed apoptosis probably arise as the consequence of mitotic catastrophe (Vitale et al., 2011). Thus, the resolution of accumulated DNA damage in ISCs and progenitors is critical in their capabilities for epithelial regeneration.

Generally, new drug development, including radioprotectors, is a costly and time-consuming process. Therefore, an alternative strategy to repurpose approved drugs with radioprotective effects could be promising. Disulfiram (DSF), also known as Antabuse, has been used as a treatment for alcoholism since it was approved by the FDA in 1948 (Johansson, 1992). The radioprotective properties of DSF have been extensively observed. Several groups have reported that DSF alleviates radiation-induced oxidative stress and DNA damage *in vitro* and *in vivo* (Gandhi et al., 2003; Nemavarkar et al., 2004). Moreover, the thiol compound diethyldithiocarbamate (DTC) is an obligatory intermediate in the metabolism of disulfiram and DTC has been reported to protect mice against a lethal dose of X-ray irradiation (Stromme and Eldjarn, 1966).

In the present study, we explored whether DSF protects against intestinal injury induced by high single-dose irradiation in a mouse model. Our results revealed that DSF decreased DNA damage accumulation in intestinal epithelium crypt cells and enhanced ISCs survival and crypt regeneration, thus ameliorating intestinal injuries and improving the survival of mice after lethal irradiation. Interestingly, DSF treatment did not inhibit radiation-induced apoptosis of crypt cells at the early phase but decreased delayed apoptosis in crypt cells. Our data suggested that DSF may be a promising protectant in the prevention of radiation-induced intestinal injury.

## Methods

### Mice and Treatment

The procedures for all animal experiments were approved by the Ethics Committee of PLA Rocket Force Characteristic Medical Center and were performed in accordance with the Guide for the Care and Use of Laboratory Animals. Male C57BL/6 mice aged 6–8 weeks were purchased from SPF Biotechnology Co., Ltd. (Beijing, China). Lgr5-EGFP-IRES-cre-ERT2 mice (The Jackson Laboratory, United States) were crossed with ROSA26-Tdtomato mice (The Jackson Laboratory, United States) to generate Lgr5-EGFP-IRES-cre-ERT2; ROSA26-Tdtomato heterozygous mice. Likewise, Bmi1-CreER mice (The Jackson Laboratory, United States) were crossed with R26R-EYFP mice (The Jackson Laboratory, United States) to generate Bmi1-CreER; R26R-EYFP mice heterozygous mice. PUMA knockout (PUMA KO) mice were kindly provided by Professor Cong (Academy of Military Medical Sciences, China). For the *in vivo* experiments, DSF (Tetraethylthiuram disulfide, 86720-50G, Sigma-Aldrich, United States), dissolved in vehicle (5% dimethyl sulfoxide, 5% Tween-80 and 90% saline), was treated to mice 1 h before irradiation at a dose of 100 mg/kg. For the *in vitro* experiments*,* DSF was dissolved in dimethyl sulfoxide (DMSO) to create concentrated stock solutions at a concentration of 50 mM and stored at −20°C.The stock solution was diluted in culture medium at the time of use. DTC (Ditiocarb sodium, HY-B1637, MedChemExpress, United States), dissolved in vehicle was injected intraperitoneally into mice 1 h before irradiation at a dose of 200 mg/kg. The animals were randomly assigned to different treatment groups. For survival experiments, at least 10 mice/groups were used and at least 3 mice/groups were used for other experiments.

### Cell Culture

Human intestinal epithelial cell (HIEC) line was purchased from Eallbio Life Sciences (Beijing, China). Cells were cultured in Dulbecco’s modified Eagle’s medium (DMEM, Gibco-Invitrogen, United States) supplemented with 10% fetal bovine serum (FBS) and 1% penicillin/streptomycin at 37°C under 5% CO_2_ incubator.

### Radiation Protocols

X-ray generated by the Precise linear accelerator (VWAT Elekta, Sweden) in the Radiation Centre (PLA Rocket Force Characteristic Medical Center, Beijing) was used as the source of radiation and radiation was delivered with a 6 MeV linear accelerator. For total body irradiation (TBI), mice were irradiated at a dose rate of 0.6 Gy/min. For abdominal irradiation, a 2 cm area of the anesthetized mouse containing the gastrointestinal tract was irradiated, thus shielding the upper thorax, head, neck, lower extremities and upper extremities as well as protecting a portion of the bone marrow by using lead plate.

### Histology Analysis

The proximal small intestines were gathered and fixed after the mice were sacrificed 3.5 days after irradiation. Paraffin-embedded sections (4 µm) were stained with hematoxylin-eosin (H&E) for observation and histological analysis. Normal mitosis contains condensed chromosomes that show even and symmetrical separation and alignment. Aberrant mitosis contains condensed chromosomes with multipolar spindles, lagging or misaligned chromosomes, anaphase bridges, or micronuclei (Leibowitz et al., 2011). Eight intestinal circumferences were randomly selected from each mouse to count regenerated crypts.

### Immunohistochemistry

Briefly, 4-μm paraffin-embedded sections were subjected to deparaffinization, antigen retrieval by sodium citrate solution, blocking with goat serum and antibody incubation. The following antibodies were used: anti-BrdU (ab6326, Abcam, United Kingdom); anti-phospho-histone H3 (9701S, CST, United States); anti-cleaved caspase-3 (9664S, CST, United States) and anti-γH2AX (05636, Merck Millipore, Germany). For 5-bromo-2′-deoxyuridine (BrdU) IHC, BrdU (B5002-5G, Sigma–Aldrich, United States) was administered to mice by intraperitoneal injection at a dose of 120 mg/kg 2 h prior to sacrifice. The viability of a surviving crypt was confirmed by incorporation of BrdU into five or more epithelial cells within each regenerative crypt. Eight intestinal circumferences were randomly selected from each mouse to count and positive cells were counted in 5 crypts in each intestinal circumference.

### Terminal Deoxynucleotidyl Transferase dUTP Nick-End Labeling Assay

Apoptosis was assessed in small intestinal sections from mice post radiation by using a TUNEL apoptosis assay kit (C1098, Beyotime, China). The detection method was performed according to the manufacturer’s instructions. The number of TUNEL positive cells per crypt was calculated in more than 50 crypts per mouse.

### Lineage Tracing Assay

To examine the contribution of intestinal stem cells to tissue regeneration and label the Lgr5^+^ ISC and the Bmi1^+^ ISC lineages, Lgr5-EGFP-IRES-cre-ERT2; ROSA26-Tdtomato mice and Bmi1-CreER; R26R-EYFP mice were injected intraperitoneally with tamoxifen in corn oil, and intestinal tissue was harvested on day 7 post-IR. The intestine was cut open and fixed in 4% formaldehyde. After cleaning the intestine, it was embedded in 4% agarose, and a 150 μm thick section was prepared using a vibrating sectioning mechanism (5100 plus, Campden, Germany). The experimental method is described in detail (Snippert et al., 2011). Lineage generation efficiency was quantified by calculating the GFP^+^ or YFP^+^ villi arising from Lgr5^+^ or Bmi1^+^ ISCs.

### Isolation of Intestinal Crypt Cells

The method of isolating intestinal crypts has been previously described (Mahe et al., 2013). Briefly, a segment of proximal intestine representing a 10-cm region from 2 to 12 cm distal to the pyloric sphincter was collected for crypt isolation. After flushing with ice-cold PBS, the small intestines were chopped into small pieces and then placed into cold DPBS containing 2 mM EDTA for 30 min. The epithelial layer was liberated from the stroma using a mixer, and the stroma was then discarded. The solution was filtered through a 70 μm strainer to remove the villus fraction and collect the crypt fraction.

### Western Blot Assay

Isolated small intestinal crypt cells were collected at 0.5, 4 and 24 h following TBI. In brief, protein lysis, quantification, electrophoresis, membrane transfer, blocking, antibody incubation and visualization using a BIO-RAD ChemiDoc MP imaging system (California, United States) were performed. The following antibodies were used: anti-γH2AX (ab2893, Abcam, United Kingdom) and anti-Vinculin (A01207, BOSTER, China).

### Immunofluorescence Assay

HIECs were seeded onto glass bottom cell culture dishes (801002, NEST, China), and pretreated with or without 0.05 μM DSF for 12 h before 6 Gy radiation. Cells were collected at indicated time points (0, 0.5, 1, 2, 4, 12 and 24 h after radiation), fixed in 4% paraformaldehyde, permeabilized with 0.1% Triton X-100, blocked with goat serum, and incubated with anti-γH2AX antibody (05636 Merck Millipore, Germany) overnight at 4°C, followed by incubation with Alexa Fluor 488 (A0428, Beyotime, China), Hoechst (C1026, Beyotime, China) counterstaining and observed by confocal microscopy (LSM-700, ZEISS, Germany). The γH2AX-focis were analyzed from the images of IF staining by using ImageJ software 1.8.0 (National Institutes of Health, United States).

### Statistical Analysis

GraphPad Prism 8.0 (GraphPad Software, San Diego, California) was used for statistical analyses. All data were obtained from at least two repetitions and were presented as the mean ± SEM unless otherwise specified. Comparisons between groups were performed using an unpaired two-tailed Student’s t-test. Log-rank analysis was performed for survival analysis. Asterisks represent the *p* values as follows: *p < 0.05, **p < 0.01, ***p < 0.001 and ****p < 0.0001. *p* < 0.05 was considered statistically significant.

## Results

### DSF Improves Survival and Prevents Intestinal Injury in Lethally Irradiated Mice

**FIGURE 1:**
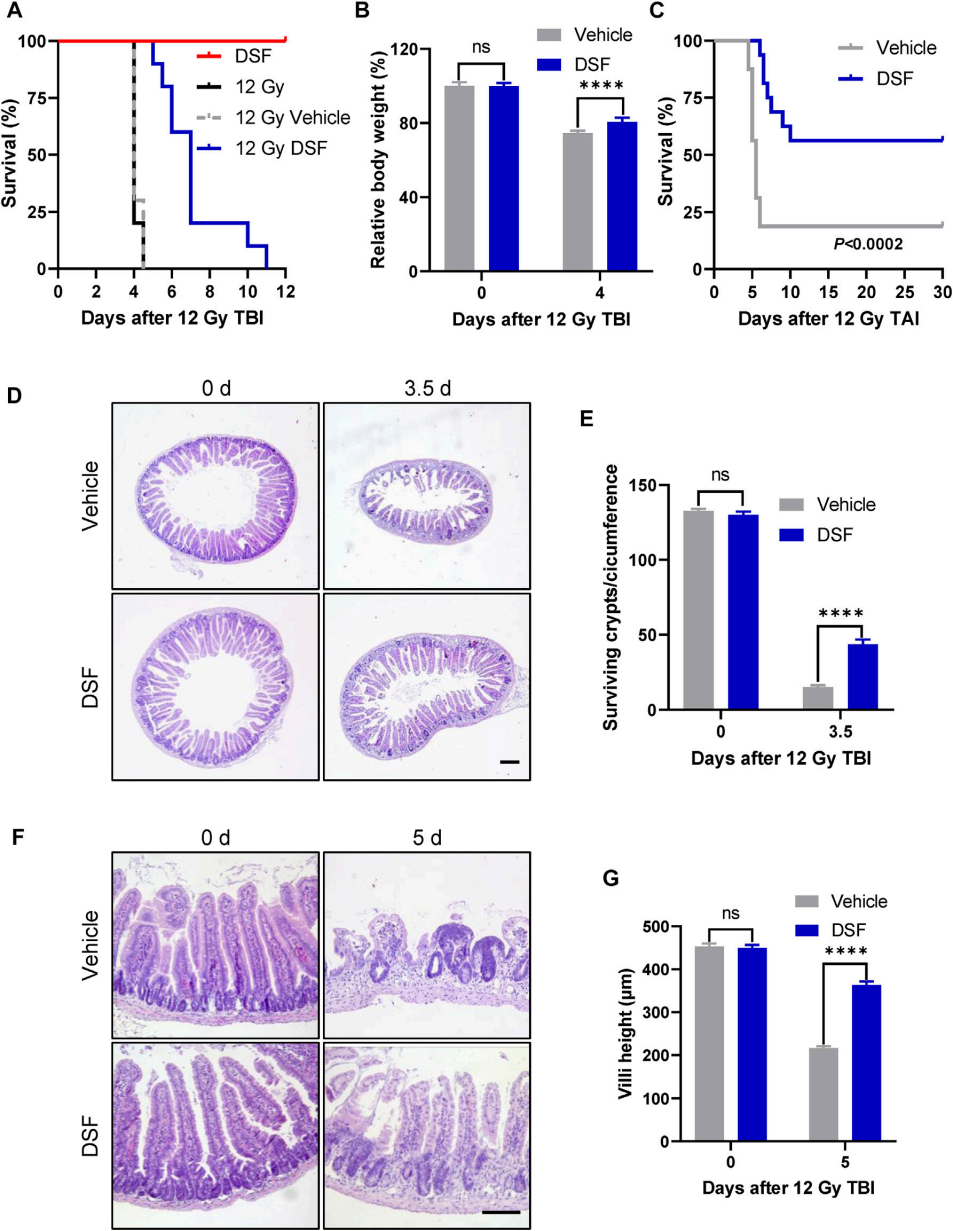
DSF protects against irradiation-induced lethal intestinal injury. Mice were intraperitoneal injected with 100 mg/kg DSF or vehicle (5% DMSO and 5% Tween-80 and 90% saline) 1 h prior to 12 Gy irradiation. **(A)** Survival curves of no treatment, vehicle, DSF- treated C57BL/6J mice (*n* = 10) after 12 Gy TBI and DSF- treated C57BL/6J mice (*n* = 10) without radiation. *p* < 0.0001, vehicle versus DSF treatment after 12 Gy TBI, by unpaired, two-tailed Student’s t-test. **(B)** Relative body weight on 4 days after TBI. **(C)** Survival curves of vehicle or DSF-treated C57BL/6J mice (n = 16) after 12 Gy of TAI. **(D)** Representative images of H&E-stained intestinal sections with the indicated treatment at 3.5 days. Scale bars = 200 μm. **(E)** Quantitative analysis of surviving crypts in **(D)**. **(F)** Representative images of H&E-stained intestinal sections with the indicated treatment at 5 days. Scale bar = 100 μm. **(G)** Quantitative analysis villus height in **(F)**. Values are means ± SEM; *n* = 3-4 mice in each group. ****p < 0.0001, vehicle versus DSF treatment, by unpaired, two-tailed Student’s t-test. DSF, disulfiram; TAI, total abdominal irradiation; TBI, total-body irradiation.

We first evaluated the radioprotective effect of DSF using a total-body irradiated mouse model. Mice were injected intraperitoneally with DSF at a dose of 100 mg/kg and then subjected to 12 Gy irradiation 1 h later. All vehicle-treated mice died within 4.5 days after irradiation with an average weight loss of 25% by 4 days, which is characteristic of lethal intestinal toxicity. In contrast, DSF-treated mice died within 5–11 days after irradiation with the average survival time prolonged from 4 to 7 days and less weight loss (Figures 1A,B). To exclude the interference of the effects of IR-induced hematopoietic failure on death, we used total-abdominal irradiation (TAI) model. After abdominal exposure to 12 Gy radiation, 81.25% (13/16) of the vehicle-treated mice died within 6 days, while 56.3% (9/16) of the DSF-treated mice survived more 30 days (Figure 1C). We further assessed the histological changes in the small intestine following radiation by H&E staining. The 12 Gy radiation exposure resulted in a decrease of approximately 90% surviving crypts at 3.5 days, whereas DSF treatment increased the percentage of surviving crypts by approximately 3-fold compared to the vehicle-treated group (Figures 1D,E). Vehicle alone has no radioprotection to the mouse small intestine (Supplementary Figure S1). Oral DSF pretreatment also increased regenerated crypts after irradiation (Supplementary Figure S2). In addition, DTC, which is a thiol metabolite of DSF, protected intestinal crypt cells after irradiation (Supplementary Figure S3). The average villus height in intestinal sections shortened to 48% of the unirradiated villi at 5 days after TBI, while DSF pretreatment increased villus height to nearly 81% (Figures 1F,G). These findings suggested that DSF protects against radiation-induced intestinal injury.

### DSF Enhances Intestinal Crypt Regeneration After Irradiation

**FIGURE 2:**
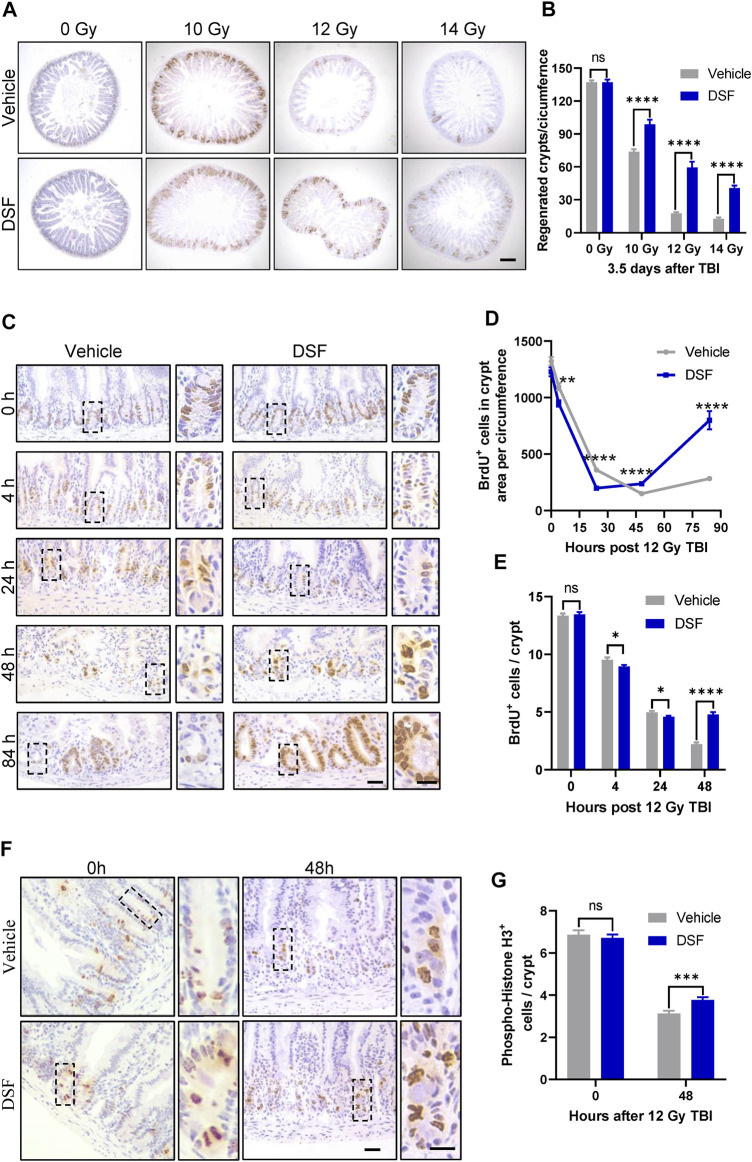
DSF enhances crypt regeneration after irradiation. Mice were intraperitoneal injected with 100 mg/kg DSF or vehicle 1 h prior to 12 Gy TBI. **(A)** Representative images of BrdU IHC of intestinal sections at 3.5 days after 10, 12 and 14 Gy TBI. Scale bar = 200 μm. **(B)** Quantitative analysis of regenerated crypts per circumference in **(A)**. **(C)** Representative images of BrdU IHC intestinal sections at the indicated times after 12 Gy TBI. Scale bar = 25 μm (left) and 10 μm (right). **(D)** Quantitative analysis of BrdU^+^ cells in crypts area per circumferences in **(C)**. **(E)** Quantitative analysis of BrdU^+^ cells per crypts in **(C)**. **(F)** Representative images of phospho-histone H3^+^ IHC in crypts at 48 h after 12 Gy TBI. Scale bar = 25 μm (left) and 10 μm (right). **(G)** Quantitative analysis of phospho-histone H3^+^ cells per crypts in **(F)**. Values are means ± SEM; n = 3-4 mice in each group. *p < 0.05, **p < 0.01 and ****p < 0.0001, vehicle versus DSF treatment, by unpaired, two-tailed Student’s t-test. BrdU,5-Bromo-2′-deoxyuridine; IHC, immunohistochemistry.

The intestinal crypt compartment is the basic unit for intestinal epithelial reconstruction (Barker et al., 2012). To clarify the effects of DSF on crypt regeneration, we monitored proliferation kinetics using the BrdU pulse assay, which is a thymidine analog specifically incorporated into the DNA of S-phase proliferating cells. In the vehicle-treated group, regenerated crypts significantly decreased when mice were exposed to progressively higher doses of radiation. In the DSF-treated group, regenerated crypts were increased by 134% (10 Gy), 328% (12 Gy) and 315% (14 Gy), respectively. (Figures 2A,B). These results suggested that DSF protects against RIII in mice exposed to a wide range of irradiation doses. A dose of 12 Gy was used in the subsequent experiments. We continued to use a BrdU incorporation assay to explore the regeneration of crypts from the start of irradiation to 84 h later (Figures 2C–E). Crypt proliferation in vehicle-treated mice was decreased by nearly 89% (from 1322 to 150) at 48 h after TBI followed by the appearance of regenerated crypts at 84 h. Interestingly, compared to the vehicle group, crypt proliferation in DSF-treated mice was inhibited within 24 h, but then it gradually recovered until exceeded at 48 h, reaching nearly 3 times of the crypt proliferation in the control group at 84 h. We further analyzed the effect of DSF on cellular proliferation by phospho-histone H3 staining, which reaches a maximum during mitosis (Ribalta et al., 2004). Forty-8 h after irradiation, there were more phospho-histone H3^+^ cells in the DSF group than in the vehicle group (Figures 2F,G). These data demonstrated that DSF alters intestinal proliferation kinetics in response to radiation, which suppresses crypt regeneration in the early phase and promotes crypt regeneration in the later phase.

### DSF Protects Intestinal Stem Cells

**FIGURE 3:**
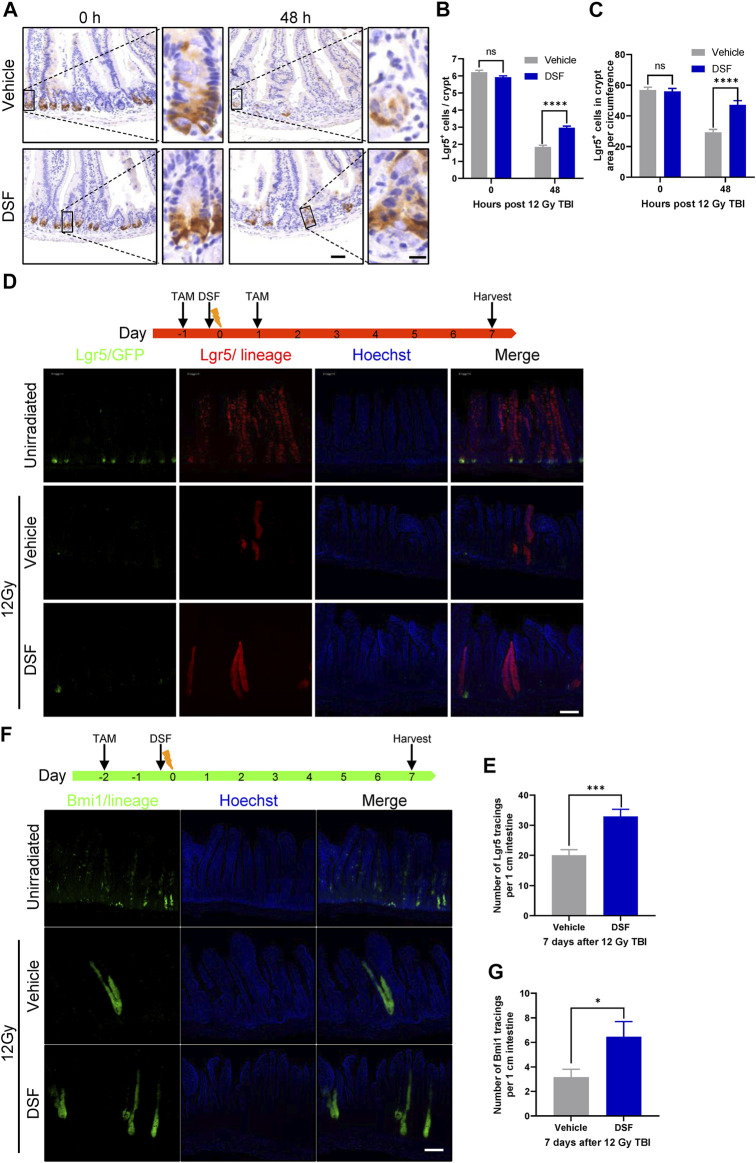
DSF protects intestinal stem cells. Mice were intraperitoneal injected with 100 mg/kg DSF or vehicle 1 h prior to 12 Gy TBI. **(A)** Representative images of Lgr5 cells by GFP staining in the intestines at 0 and 48 h. Scale bar = 50 μm (left) and 10 μm (right). **(B)** Quantitative analysis of Lgr5-positive cells per crypt in **(A)**. **(C)** Quantitative analysis of Lgr5-GFP cells in crypt area per circumference in **(A)**. **(D)** Two doses (100 mg/kg) of tamoxifen were injected intraperitoneally into Lgr5-eGFP-IRES-CreERT2; Rosa26-TdTomato mice at the 24 h before and after 12 Gy irradiation. Tissue was harvested on 7 days after 12Gy TBI. Representative confocal images of Lgr5 lineage regenerative response in the jejunum. Scale bar = 200 μm. **(E)** Quantitative analysis of Lgr5 tracings per 1 cm intestine in **(D)**. **(F)** Bmi1-CreER; Rosa26-YFP mice were intraperitoneal injected with 200 mg/kg tamoxifen at 0 and 48 h before 12 Gy irradiation. Tissue was harvested on 7 days after 12Gy TBI. Representative confocal images of Bmi1 lineage regenerative response in the jejunum. Scale bar = 200 μm. **(G)** Quantitative analysis of Bmi1 tracings per 1 cm intestine in **(F)**. Values are means ± SEM; n = 3-5 mice in each group. *p < 0.05, ***p < 0.001, ****p < 0.0001, vehicle versus DSF treatment, by unpaired, two-tailed Student’s t-test. TAM, tamoxifen.

The leucine-rich repeat-containing G-protein coupled receptor 5 (Lgr5) is a marker of ISCs, and the Lgr5^+^ ISCs are indispensable for IR-induced intestinal epithelium regeneration (Metcalfe et al., 2014). To assess the effect of DSF on Lgr5^+^ ISCs after irradiation, we used Lgr5-EGFP-IRES-creERT2 mice in which the allele was knocked in by integrating an enhanced green fluorescent protein (EGFP)-IRES-creERT2 cassette after Lgr5 (Barker et al., 2007). Radiation reduced the loss of more than 70% Lgr5-GFP cells by 48 h, which was significantly suppressed in the DSF group (Figures 3A,B). At the same time, the fraction of Lgr5^+^ crypts increased by over 30% in the DSF-treated group (from 52% to 84% of unirradiated level, Figure 3C). To further clarify whether DSF exerts radioprotection *via* Lgr5^+^ ISC-mediated epithelial regeneration, we generated Lgr5-eGFP-IRES-CreERT2; Rosa26-TdTomato mice by crossing Lgr5-eGFP-IRES-CreERT2 mice and Rosa26-TdTomato mice (carrying a conditional Tdtomato allele at the Rosa 26 locus that is activated by Cre recombinase) reporter mice. We injected these mice with tamoxifen to induce recombination in Lgr5-expressing cells 24 h before and after 12 Gy TBI. The mice pretreated with DSF produced 1.6 times more progeny than the vehicle group by 7 days of lineage tracing (Figures 3D,E). Similarly, we analyzed Bmi1 cells, which are considered another type of stem cell (Sangiorgi and Capecchi, 2008), by lineage tracing in Bmi1-CreER; Rosa26-YFP mice (Figure 3F). We observed mild protection by DSF against the loss of progeny generation by Bmi1^+^ cells (Figure 3G). Taken together, these results suggested that DSF protects intestinal stem cells.

### DSF Pretreatment has no Effect on Early Phase Apoptosis but Mitigates the Delayed Apoptosis of Crypt Cells After Irradiation

**FIGURE 4:**
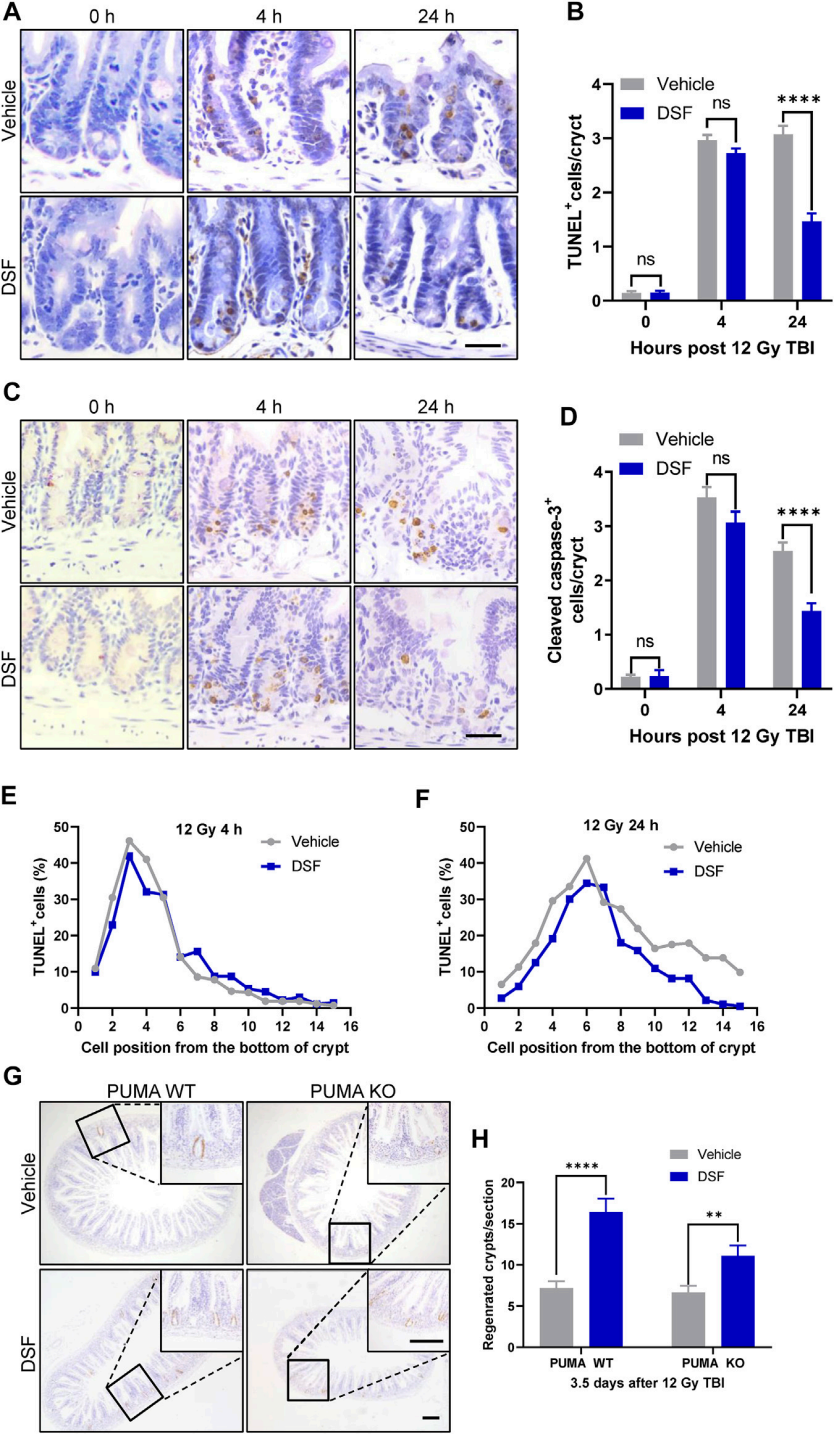
DSF pretreatment has no effect on early phase apoptosis but mitigates the delayed apoptosis of crypt cells after irradiation. Mice were intraperitoneal injected with 100 mg/kg DSF or vehicle 1 h prior to 12 Gy irradiation. **(A)** Representative images of TUNEL staining. Scale bar = 20 μm. **(B)** Quantitative analysis of TUNEL-positive cells in **(A)**. **(C)** Representative images of cleaved caspase 3 IHC. Scale bar = 20 μm. **(D)** Quantitative analysis of cleaved caspase3-positive cells in **(C)**. **(E)** Fractions of TUNEL-positive crypt cells at 4 h based on cell positions in **(A)**. **(F)** Fractions of TUNEL-positive crypt cells at 24 h based on cell positions in **(A)**. **(G)** Representative images of BrdU IHC in small intestine of PUMA WT and PUMA KO mice 3.5 days after 12Gy TBI. Scale bar = 200 μm (top) and 200 μm (bottom). **(H)** Quantitative analysis of regenerated crypts per circumference in **(G)**. Values are means ± SEM; n = 3-4 mice in each group. **p < 0.05 and ****p < 0.0001, vehicle versus DSF treatment, by unpaired, two-tailed Student’s t-test.

Intestinal epithelium apoptosis induced by radiation occurs in two distinct phases as follows: radiation exposure rapidly activates p53-PUMA-dependent apoptosis *via* the intrinsic pathway, which peaks 3–6 h following radiation; and a delayed wave of p53-independent apoptosis emerges at 24 h after irradiation (Kirsch et al., 2010). Therefore, we determined crypt cell apoptosis by TUNEL and cleaved caspase-3 (CC3) staining 4 and 24 h after 12 Gy TBI (Figures 4A–F). Unexpectedly, apoptotic cells (marked by TUNEL or CC3 staining) in DSF-treated mice occurred to a similar degree at 4 h after radiation compared to vehicle-treated mice. However, 24 h after radiation, the total number of apoptotic crypt cell in the DSF-treated group was reduced by half compared to that in the control group. More specifically, apoptosis at cell positions 4, 5 and 6 above the crypt base was suppressed by 36, 10 and 16%, respectively, in DSF-treated mice at 24 h. To further exclude the possibility that DSF exerts radioprotective effects on intestinal recovery and is involved in preventing early intrinsic apoptosis after irradiation, PUMA KO mice were treated with DSF or vehicle and were subjected to 12Gy TBI. The results showed that DSF significantly promoted crypt regeneration, nearly doubling the crypt (Figures 4G,H). These results suggested that DSF promotes intestinal regeneration after irradiation through additional mechanisms that are not directly related to suppression of early intrinsic apoptosis.

### DSF Alleviates DNA Damage Accumulation and Decreases Aberrant Mitosis

**FIGURE 5:**
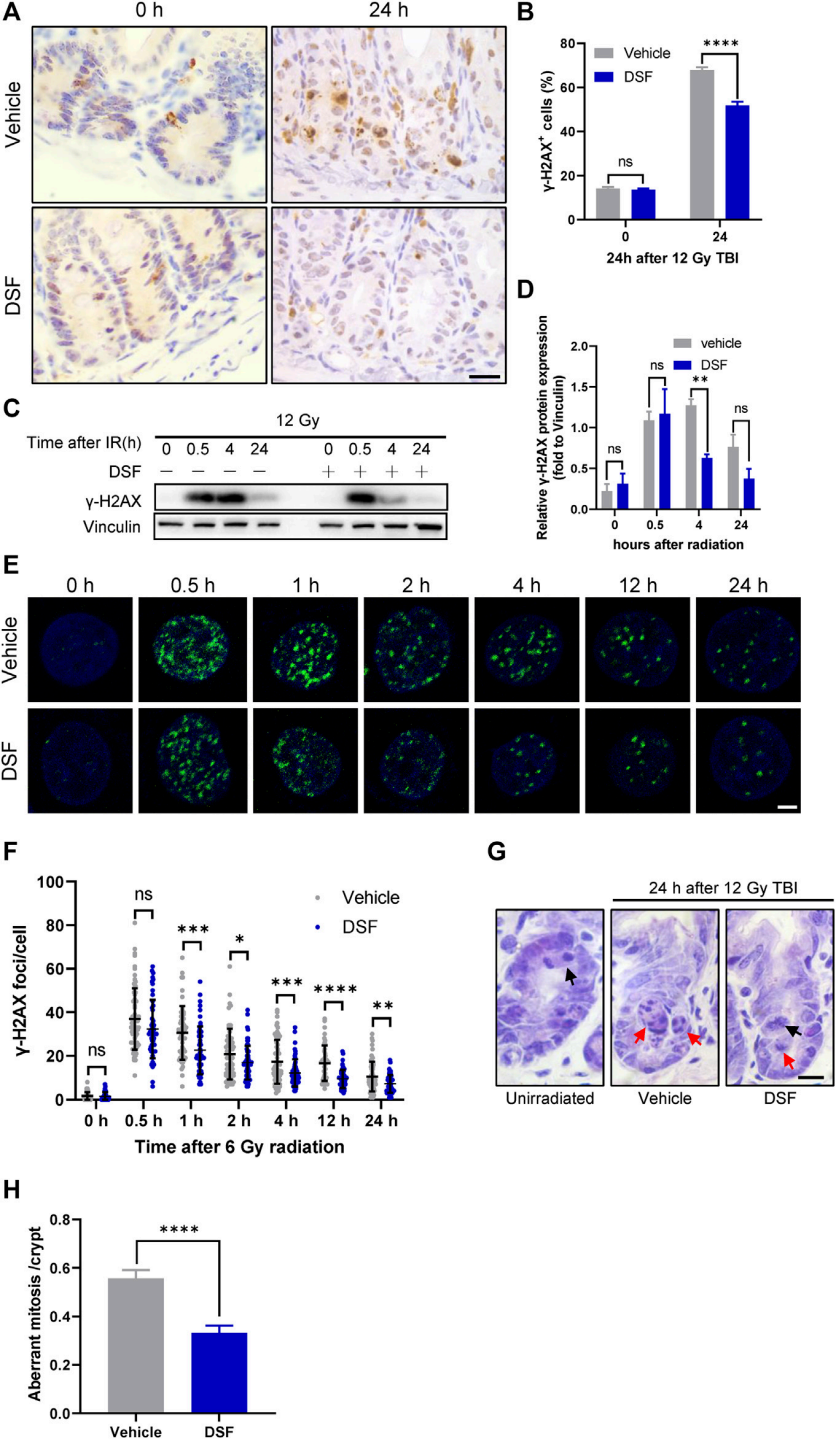
DSF alleviates DNA damage accumulation and decreases aberrant mitosis**.** Mice were intraperitoneal injected with 100 mg/kg DSF or vehicle 1 h prior to 12 Gy irradiation. **(A)** Representative images of γH2AX IHC at 0 and 24 h. Scale bar = 20 μm. **(B)** Quantitative analysis of γH2AX-positive crypt cells (%) in **(A)**. **(C)** Western blotting detection of γH2AX protein expression of small intestinal crypt cells. **(D)** The protein levels of γH2AX were detected by western blot assays at indicated time points after radiation with vinculin as the internal control. **(E)** Representative IF images showing γ-H2AX foci in HIEC cells pretreated with or without 0.05 μM DSF at indicated time points after 6 Gy radiation. γH2AX foci are shown in green. Scale bar = 5 μm. **(F)** Quantitative analysis of γH2AX foci per cell. **(G)** Representative images of H&E-stained intestinal sections at 24 h after 12Gy radiation. Examples of normal and aberrant mitosis are indicated by black arrows and red arrows respectively. Scale bar = 10 μm. **(H)** Quantitative analysis of aberrant mitoses per crypts in **(F)**. Values are means ± SEM; n = 3-4 mice in each group. *p < 0.05, **p < 0.05, ****p < 0.0001 and ****p < 0.0001, vehicle versus DSF treatment, by unpaired, two-tailed Student’s t-test.

Incomplete repair of IR-induced DNA DSBs leads to the accumulation of DNA lesions and genome instability, and aberrant mitosis may occur when the cell cycle reinitiates after irradiation (Vitale et al., 2011). Delayed phase (24 h) γH2AX foci are considered a surrogate for unrepaired DSBs(Hua et al., 2012). To examine unsolved DNA lesions at 24 h post irradiation, we first detected DNA DSBs by γH2AX staining and found that DSF dramatically decreased the proportion of γH2AX-positive crypt cells in irradiated (Figures 5A,B). To further clarify the effect of DSF on IR-induced DNA damage repair kinetics, we isolated intestinal crypts at different time points after irradiation and analyzed γH2AX expression using immunoblotting. At 0.5 h after irradiation, the rapidly elevated expression of γH2AX was similar between the DSF treatment group and the control group. At 4 h after irradiation, however, the γH2AX level of the DSF treatment group was significantly lower than that of the control group (Figures 5C,D). In addition, the DSBs in the HIECs at different time points after irradiation were detected through γH2AX immunofluorescence staining. γH2AX foci formation occurs rapidly in irradiated cells, reaching a maximum at 0.5 h and then declining gradually within 24 h following irradiation (Figures 5E,F). We observed the similar number of γH2AX foci between two groups at 0.5 h after radiation, however, the number of γH2AX foci in cells treated with DSF at a concentration of 50 nM, which had no cytotoxic effect (Supplementary Figure S4), were significantly lower than in vehicle-treated cells at each subsequent time point. These results indicate that DSF protects crypt cells by facilitating DNA repair rather than directly suppressing DNA DSBs induced by irradiation. Moreover, the effect of DSF on the mitotic progression of intestinal crypt cells was examined by H&E staining (Figures 5G,H). Twenty-4 h after 12 Gy TBI, most of the mitotic crypt cells were aberrant because the chromosomes were arranged in an asymmetrical manner (Kirsch et al., 2010; Leibowitz et al., 2011), whereas DSF treatment suppressed aberrant mitosis by 40% compared to the vehicle group. These findings provide evidence that DSF prevents accumulation of DNA damage in intestinal crypt cells, thereby avoiding genome instability.

## Discussion

The small intestine is particularly vulnerable to planned radiation exposure during pelvic and abdominal radiotherapy in cancer treatment as well as unintended radiation exposure during a nuclear accident. Unfortunately, no safe and effective prophylactics or therapeutics have been approved at present for RIII (Singh and Seed, 2017). In the present study, we provided *in vivo* evidence that the FDA-approved drug DSF confers a protective effect on RIII. Our data revealed that DSF pretreatment prolonged the survival time and attenuated weight loss in C57BL/6J mice exposed to lethal TBI. Histologically, DSF promoted crypt survival and accelerated epithelium regeneration after whole-body exposure. Van Bekkum et al. reported that animals injected with DTC, but not DSF, is effectively protected against irradiation (Van Bekkum, 1956). Further research suggested that the antiradiation effect of DSF was mediated by its major metabolite, DTC. DTC gave rise to high concentrations of free, unmetabolized thiol, whereas no free thiol was found after the administration of disulfiram (Stromme and Eldjarn, 1966). However, we found that both DSF and DTC significantly enhanced crypt regeneration. The contradiction with the previous study may be due to the variation in the administration method, dosage and animal model used. Oral DSF was also effective for RIII, but the effect was not as good as intraperitoneal injection at 1 h of pretreatment time, which might be explained by its poor oral bioavailability. Because the radioprotective efficiency of agents may vary between species and strains (Williams et al., 2012), the development of DSF as radioprotector requires further studies in large animal models, including canines, minipigs and nonhuman primates. The antitumor effects of DSF have recently been reported in multiple studies (Camp et al., 1981), and further study will be needed to determine whether DSF treatment is also protective against conventionally fractionated radiotherapy, especially when used for spontaneously growing abdominal and pelvic tumors.

ISC depletion is the key event in the pathophysiology of radiation-mediated intestinal toxicity. Mitotically active Lgr5^+^ cells reside at the base of the crypt and can differentiate into all types of cells that make up the small intestine epithelium (Sato et al., 2009; Hua et al., 2012). Metcalfe et al. revealed that Lgr5^+^ crypt cells were indispensable for radiation-induced intestinal regeneration (Metcalfe et al., 2014). Moreover, survival of Lgr5^+^ CBCs at 2 days after irradiation predicts crypt regeneration at 3.5 days and lethality from gastrointestinal syndrome (Hua et al., 2012). Consistently, we found that DSF treatment attenuated the loss of Lgr5^+^ ISCs *in vivo* 2 days after 12 Gy irradiation. Upon tamoxifen treatment in Lgr5-eGFP-IRES-CreERT2; Rosa26-TdTomato mice, Lgr5^+^ ISCs in the DSF-treated group were significantly more efficient at producing progeny than those in the vehicle-treated group by 7 days of lineage tracing following 12 Gy TBI. Our data clearly showed that DSF-mediated radioprotection is closely related to the population of surviving Lgr5^+^ ISCs in the crypts of irradiated mice. Bmi1^+^ cells are located at position +4 relative to the crypt base and are postulated to constitute a quiescent stem cell pool, which facilitates radiation-induced regeneration (Yan et al., 2012). However, subsequent studies have shown that Bmi1 is co-expressed by Lgr5^+^ stem cells (Muñoz et al., 2012), and Clevers et al. considered only Lgr5^+^ cells as bona fide ISCs and position 4 cells as descendants (Gehart and Clevers, 2019). The more robust expansion of Bmi1^+^ lineage in DSF-treated group vs. vehicle-treated control after radiation was observed in our study. We cannot rule out the possibility that DSF directly preserves Bmi1^+^ cells or that DSF indirectly enhances the mitosis of Bmi1^+^ cells derive from surviving Lgr5^+^ cells.

Death response of ISCs and intestinal progenitor cells to radiation appear to occur through two independent mechanisms, which are closely related to the proliferative status of the cell, with apoptosis occurring during interphase and mitotic death occurring upon reinitiation of the cell cycle (Leibowitz et al., 2011). The p53-PUMA intrinsic pathway plays a critical role in mediating crypt cell apoptosis at the early phase after irradiation (Leibowitz et al., 2011). Blocking PUMA-dependent apoptosis protects stem and progenitor cells, and it profoundly attenuates intestinal injury after irradiation (Qiu et al., 2008). However, we found no difference in early apoptosis of intestinal cells between DSF- and vehicle-treated irradiated mice, and DSF treatment still promoted crypt regeneration in PUMA-deficient mice. Ionizing radiation induces cellular DSBs, which rapidly activate cell cycle checkpoints and block the cell cycle to enable DNA damage repair. When the repair is not completed and reinitiates cell division, the cell is prone to undergo mitotic death. Mitotic death can initiate apoptosis, and features of delayed apoptosis have been observed in the intestinal epithelium succumbing to mitotic failure (Merritt et al., 1997). Therefore, DSF-mediated inhibition of aberrant mitosis may explain why DSF can effectively reduce delayed apoptosis 24 h after irradiation. Deficiency in cell cycle checkpoints exacerbates mitotic death with elevated DNA damage despite blocking early apoptosis (Leibowitz et al., 2011). Temporary blockade of cell cycle progression with a CDK4/6 inhibitor facilitates DNA damage repair of intestinal cells and protects against radiation-induced intestinal injury in mice (Wei et al., 2016). Correspondingly, we found that DSF treatment changes the kinetics of intestinal proliferation. DSF treatment inhibited proliferation within 24 h after irradiation and increased proliferation to a higher level than that of the control group after 48 h of irradiation. In addition, we observed fewer γH2AX-positive crypt cells in DSF-treated mice and less γH2AX foci in cultured cells 24 h following radiation, indicating that DSF reduces the accumulation of DNA lesions. These findings were consistent with previous reports, showing that DSF decreases radiation-induced DNA damage in yeast and mouse liver cells (Gandhi et al., 2003; Nemavarkar et al., 2004). Our data demonstrated that DSF reduces cell death by regulating the DNA damage response through cell cycle arrest, thereby exerting a radioprotective effect. Further research is needed to clarify which pathway for DSB repair is promoted by DSF to alleviate radiation-induced DNA damage.

## Conclusion

**FIGURE 6:**
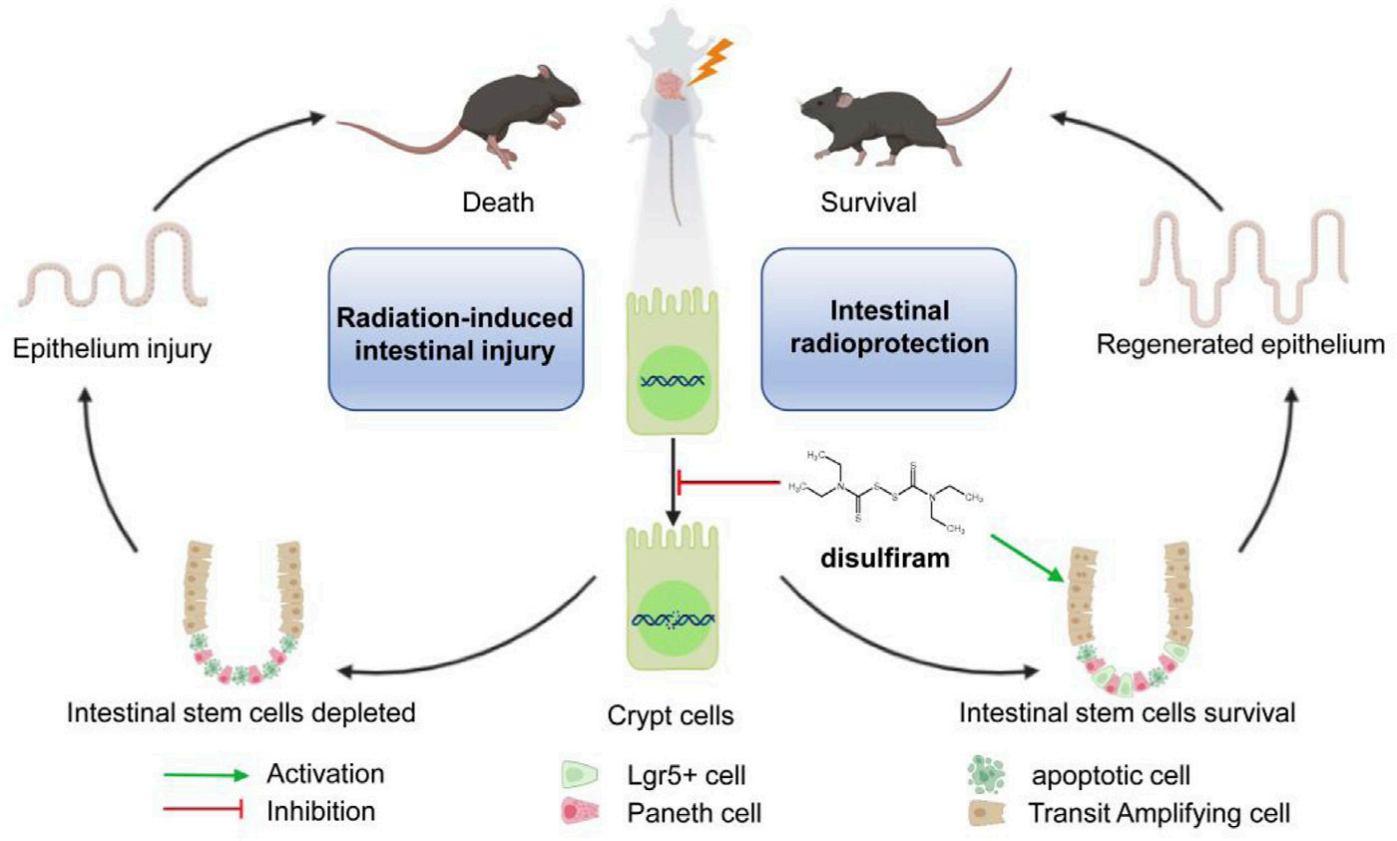
A schematic diagram summarizing the mechanism of Disulfiram protects against radiation-induced intestinal injury in mice. Ionizing radiation induces cellular DNA double-strand break. DSF reduces the accumulation of DNA lesions and protects intestinal stem cells, which further prevents intestinal injury and improves survival in lethally irradiated mice.

Our study reports the efficacy of disulfiram against lethally irradiated intestinal injury. Disulfiram reduces the DNA damage accumulation and promotes the survival of intestinal stem cells through affecting the cell cycle after irradiation (Figure 6). These results suggest a potential novel clinical application of disulfiram for intestinal injury in radiotherapy and accidental radiation exposure.

## Data Availability

The original contributions presented in the study are included in the article/Supplementary Material, further inquiries can be directed to the corresponding authors.

## Abbreviations

BrdU,5-Bromo-2′-deoxyuridine; CC3, cleavedCaspase-3; DTC, diethyldithiocarbamate; DMSO, dimethyl sulfoxide; DNA, deoxyribonucleic acid; DSB, double-strand break; DSF, disulfiram; FBS, fetal bovine serum; FDA, food and drug administration; HIEC, human intestinal epithelial cell; H&E, hematoxylin-eosin; IF, immunofluorescence; IHC, immunohistochemistry; IR, ionizing radiation; ISCs, intestinal stem cells; PUMA KO, the p53 upregulated modulator of apoptosis knockout; RIII, Radiation-induced intestinal damage; SEM, standard error mean; TAI, total abdominal irradiation; TAM, tamoxifen; TBI, total body irradiation.
