# Supplementary figures and images for: Disulfiram Protects Against Radiation-Induced Intestinal Injury in Mice
Source: Front Pharmacol. 2022 Apr 19;13:852669. doi: 10.3389/fphar.2022.852669 (PMC9061966; doi:10.3389/fphar.2022.852669)

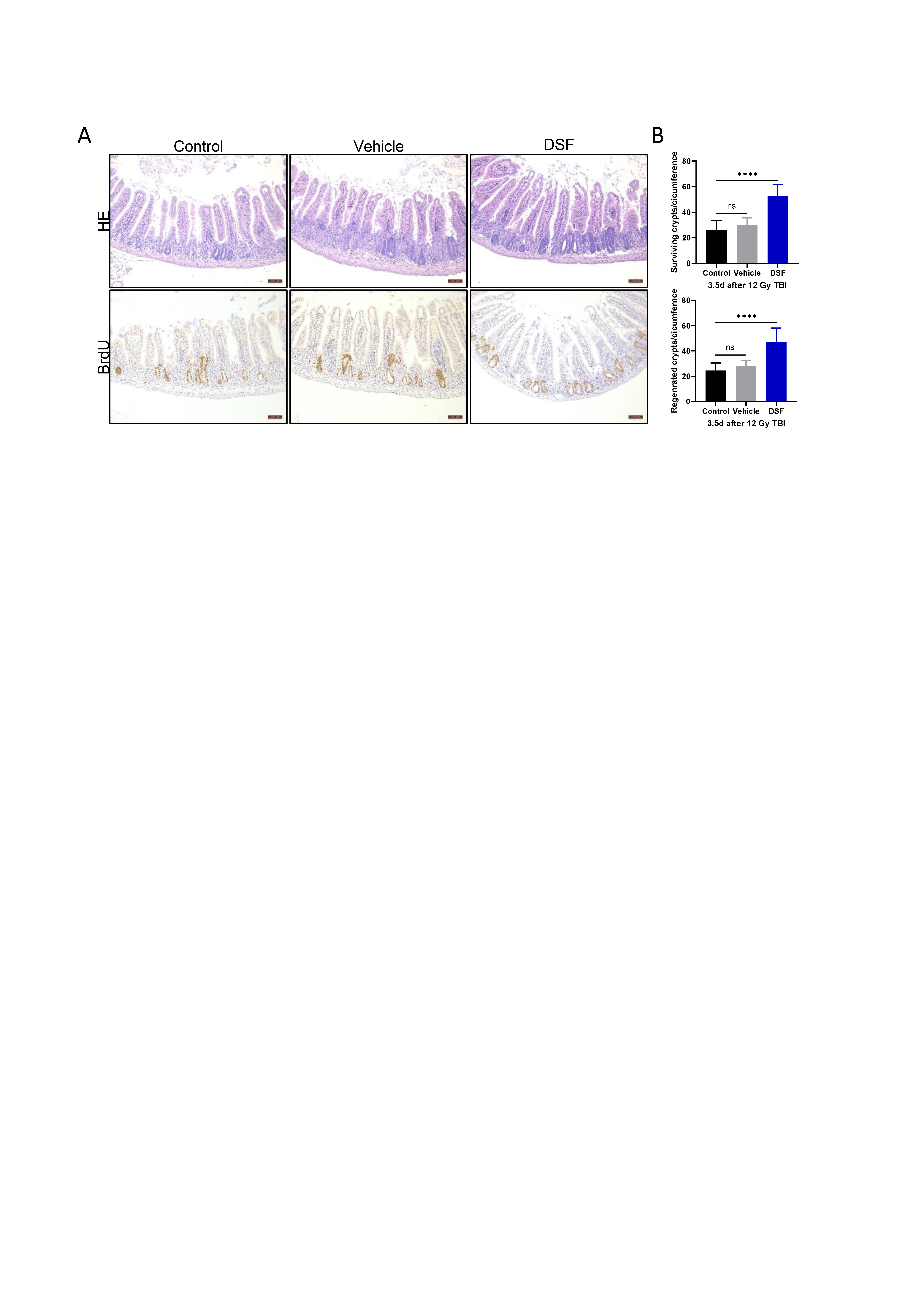

Supplement: Supplementary file 1 [file Image1.TIFF]

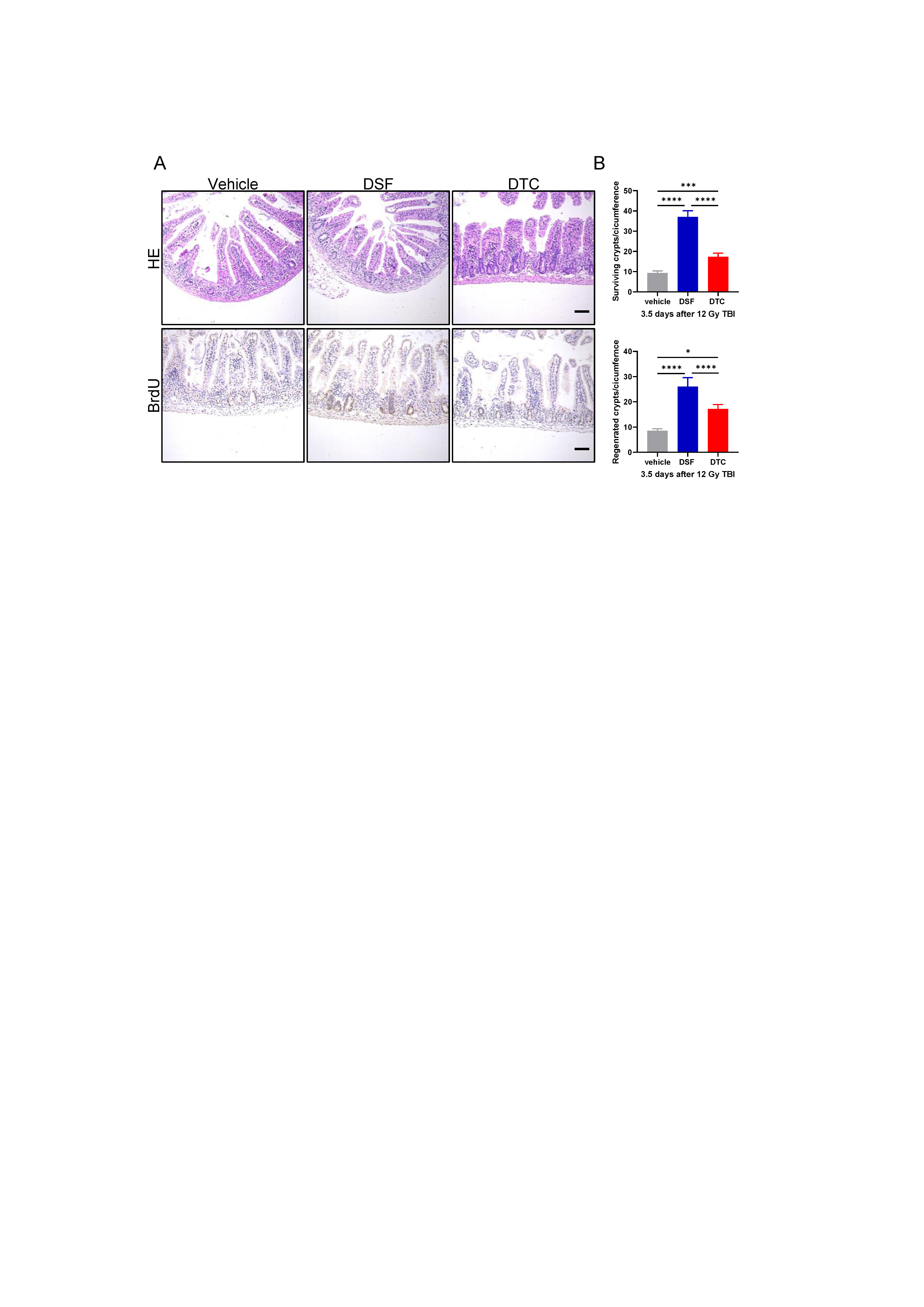

Supplement: Supplementary file 2 [file Image3.tif]

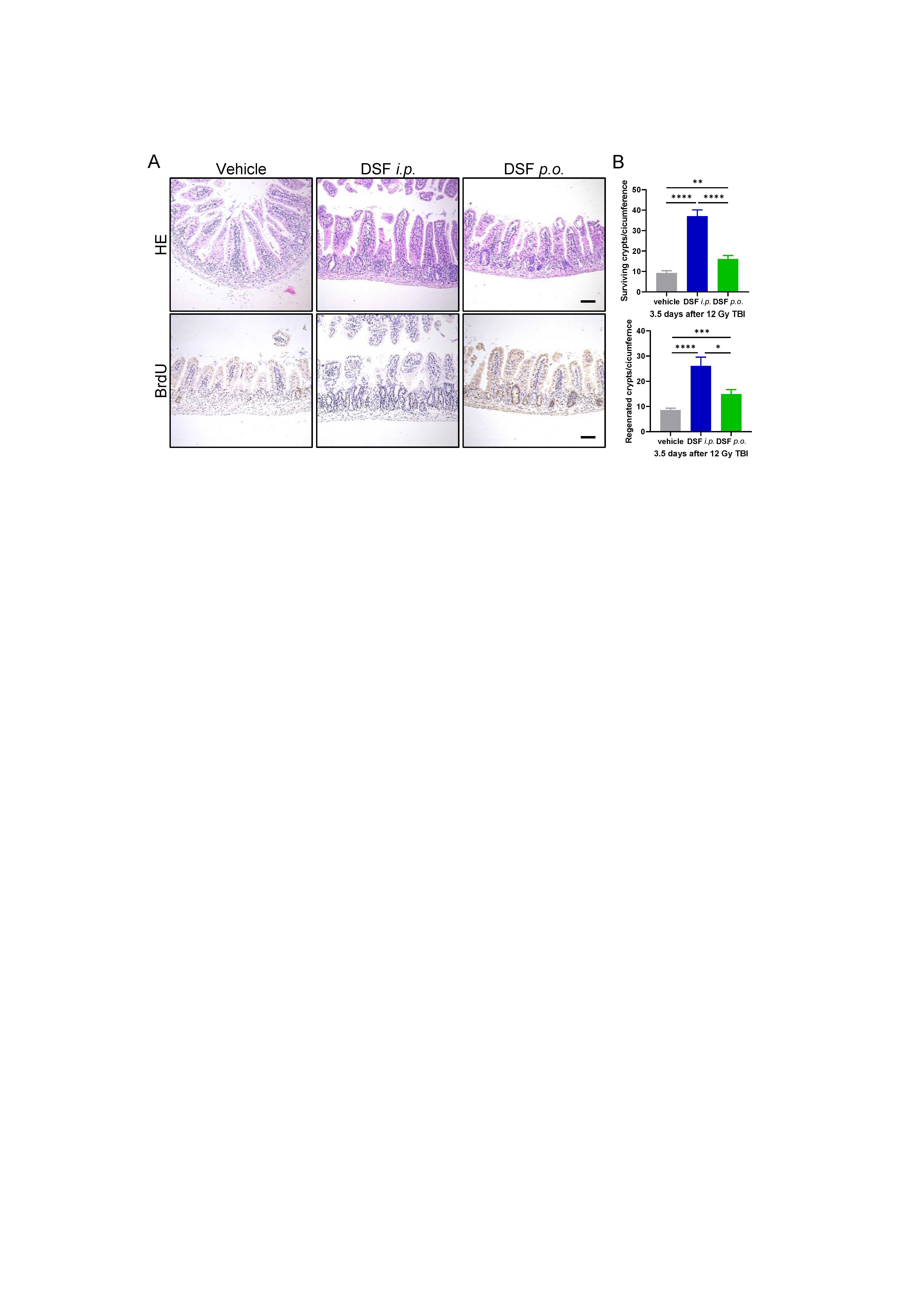

Supplement: Supplementary file 4 [file Image2.TIF]
