# Supplementary material for: Disulfiram Protects Against Radiation-Induced Intestinal Injury in Mice
Source: Front Pharmacol. 2022 Apr 19;13:852669. doi: 10.3389/fphar.2022.852669 (PMC9061966; doi:10.3389/fphar.2022.852669)
Supplement: Supplementary file 5 [file DataSheet1.docx]

**Disulfiram protects against radiation-induced intestinal injury in mice**

**Supplemental figure 1. Vehicle provided no contribution to radiation protection.** Mice were not treatment (control) or intraperitoneal injected with vehicle (5% DMSO and 5% Tween-80 and 90% saline) or 100 mg/kg DSF dissolved in vehicle 1 h prior to 12 Gy irradiation. **(A)** Representative images of H&E-stained and BrdU IHC of intestinal sections at 3.5 d after 12 Gy TBI; Scale bar = 200 μm. **(B)** Quantitative analysis of surviving crypts and regenerated crypts per circumference in **A**. Values are means ± SD; n = 3-4 mice in each group. *****p* < 0.0001, ns, non-significant. two-tailed Student’s t-test were used to determine statistical significance in **B**.

**Supplemental figure 2. Oral DSF promotes intestinal crypt regeneration following radiation.** Mice were intraperitoneally (*i.p.*) or orally (*p.o.*) administrated with 100 mg/kg DSF 1 h prior to 12 Gy irradiation. **(A)** Representative images of H&E-stained and BrdU IHC of intestinal sections at 3.5 d after 12 Gy TBI; Scale bar = 100 μm. **(B)** Quantitative analysis of surviving crypts and regenerated crypts per circumference in **A**. Values are means ± SEM; n = 3-4 mice in each group. **p* < 0.05, ***p* < 0.01, ****p* < 0.001, *****p* < 0.0001. two-tailed Student’s t-test were used to determine statistical significance in **B**. BrdU,5-Bromo-2’-deoxyuridine; DSF, disulfiram; TBI, total-body irradiation; H&E, hematoxylin-eosin.

**Supplemental figure 3: DTC protects intestinal crypt cells.** Mice were intraperitoneal injected with 100 mg/kg DSF, vehicle (5% DMSO and 5% Tween-80 and 90% saline) or 200 mg/kg DTC before 1 h prior to 12 Gy irradiation. **(A)** Representative images of H&E-stained and BrdU IHC of intestinal sections at 3.5 days after 12 Gy TBI; Scale bar = 100 μm. **(B)** Quantitative analysis of surviving crypts and regenerated crypts per circumference in **A**. Values are means ± SEM; n = 3-4 mice in each group. ****p* < 0.001 and *****p* < 0.0001. vehicle versus DSF treatment versus DTC, by unpaired, two-tailed Student’s t-test. DTC, diethyldithiocarbamate.

**Supplemental figure 4: Cytotoxicity of DSF on HIEC cells.** HIECs were treated with the indicated concentrations of DSF for 72 h. Cell viability was determined using the CCK-8 assay. Error bars indicate mean ± SD, **p* < 0.05, *****p* < 0.0001, ns, non-significant. by unpaired, two-tailed Student’s t-test.
